# Portable neuroimaging and AI: democratizing brain diagnostics

**DOI:** 10.3389/fneur.2026.1786660

**Published:** 2026-04-24

**Authors:** Jose E. Leon-Rojas

**Affiliations:** 1 Grupo de Investigación Bienestar, Salud y Sociedad, Escuela de Psicología y Educación, Universidad de Las Américas, Quito, Ecuador; 2 Escuela de Medicina, Universidad de Las Américas, Quito, Ecuador

**Keywords:** artificial intelligence, global health, health disparities, low and mid income countries, neurological disorder, nueroimaging, portable MRI, teleneurology

## 1 Introduction

Advanced neuroimaging, particularly magnetic resonance imaging (MRI), is central to modern neurological diagnosis; yet access to MRI remains profoundly unequal (1–4). Conventional scanners are expensive, infrastructure intensive, and concentrated in large urban hospitals, leaving rural and low-income regions underserved; approximately 66% of the global population does not have access to an MRI scanner (3, 4). Furthermore, low-and-middle income countries (LMICs) average approximately 1.1 MRI units per million inhabitants compared with more than 25 per million in high income countries, meaning that a majority of the global population lacks timely access to brain imaging (5). In Latin America and the Caribbean, the average is roughly 3 to 4 scanners per million people, reflecting a persistent structural inequity in neurological care (6); as a result, strokes, brain tumors, trauma, infections, and pediatric neurological disorders are frequently diagnosed late or not at all (7). Recent technological advances offer a credible path to narrowing this gap. Ultra-portable, low-cost neuroimaging devices, combined with artificial intelligence (AI) driven image reconstruction and diagnostic support, can bring brain imaging directly to the point of care, at a lower cost (5, 8). Therefore, in this opinion article I argue that the convergence of portable neuroimaging and AI represents a public health opportunity to improve neurological disease prevention at primary, secondary, and tertiary levels, particularly in underserved regions of Latin America where economical and accessibility constraints have long affected public health. Beyond technical innovation, these tools enable a rethinking of how neurological diagnostics are delivered, shifting from centralized, specialist dependent systems to more distributed and equitable models of care that fit the countries' realities rather than setting an unrealistic standard that could only be met in resource-rich settings. Therefore, this opinion goes beyond a technical review by proposing a structured, equity-oriented framework for integrating portable neuroimaging and artificial intelligence into health systems; specifically, I argue for a tiered, decentralized model in which low-field MRI and AI-supported diagnostics are included in primary and secondary care levels for triage, early detection, monitoring, and clinical decision making while complex cases are escalated to tertiary centers with specialist oversight. This approach reframes portable neuroimaging not as a substitute for high-field systems, but as a complementary public health tool designed to reduce structural inequities in access to neurological diagnostics and care in areas with limited resources such as LMICs.

## 2 Bridging the neuroimaging gap

Limited access to imaging is a major determinant of poor neurological outcomes in LMICs (6, 9). Traditional MRI systems cost well over one million US dollars and require shielded rooms, cryogenic cooling, stable power supply, and highly trained personnel; even when equipment exists, workforce shortages remain an important issue (6, 9). Many countries in Latin America and Africa have fewer than three radiologists per 100,000 inhabitants outside major cities, leading to delays in image interpretation and treatment initiation (10, 11). In this context, the challenge is not merely technological but systemic.

Portable MRI addresses these constraints by radically reducing size, power requirements, and operational complexity. Ultra-low field systems, typically operating below 0.1 Tesla, can be wheeled to the bedside, powered by a standard electrical outlet, and operated by non-specialist staff after brief training (12–14). The most widely deployed example to date is the Hyperfine Swoop system, a 0.064 Tesla scanner costing a fraction of conventional MRI and requiring no dedicated suite or cryogens (12, 15). By lowering infrastructure and staffing thresholds, such devices make advanced imaging feasible in district hospitals, community clinics, and mobile health units. Furthermore, studies have proven their validity in relevant neurological conditions (12–14); for instance, a 2021 study looking into intracerebral hemorrhage, using a 0.064T portable MRI unit, reported an 80.4% sensitivity (95%CI, 68–90) and 96.6% specificity (95%CI, 90–99) with an excellent intraclass correlation coefficient (0.955) for hematoma volumes when compared to standard imaging (13). Crucially, portability shifts the care paradigm; rather than transporting unstable patients over long distances, imaging can occur where the patient is, including intensive care units and emergency settings. This is particularly relevant in regions with geographic barriers, limited ambulance networks, and high out of pocket travel costs, all of which disproportionately affect rural and indigenous populations in LMICs (16). To facilitate interpretation of the current evidence base, Table 1 summarizes key studies evaluating portable low-field MRI across different clinical settings and indications.

**Table 1:** Representative studies evaluating portable low-field MRI in neurological conditions.

Study	Setting	Indication	Sample size	Key findings	Diagnostic performance/outcomes
Mazurek et al. 2021	Tertiary hospital (ICU, USA)	Intracerebral hemorrhage	94 patients (144 scans; 56 ICH cases)	Bedside portable MRI feasible in acute care; strong agreement in hematoma volume estimation	Sensitivity 80.4% (95% CI 68–90); specificity 96.6% (95% CI 90–99); ICC ≈ 0.96 for hematoma volume
von Danwitz et al. 2025	Tertiary stroke center	Acute ischemic stroke	17 patients (12 with ischemic lesions)	Feasible in acute stroke workflow; small infarcts (< 6 mm) may be missed	Detection: 8/12 ischemic lesions identified; 100% agreement in clinical decision-making between blinded specialists and standard imaging
Gruber et al. 2025	Multicentre tertiary pediatric centers	Hydrocephalus/ventriculomegaly	153 pediatric patients	Accurate ventricular size assessment; suitable for monitoring	Evans index: CCC ≈ 0.92 (95% CI ~0.89–0.94); FOHR: CCC ≈ 0.94 (95% CI ~0.92–0.96); strong agreement with standard MRI
Chetcuti et al. 2022	Tertiary hospital, resource-limited setting (Malawi)	Mixed neurological conditions	260 brain scans (implementation cohort)	Real-world deployment in LMIC; feasible integration into routine care	Successful bedside imaging in critically ill patients; enabled diagnosis where imaging unavailable; reduced need for patient transport; effective use by locally trained staff; integration into clinical workflow
Zeyen et al. 2025	Tertiary neuro-oncology center	Brain tumors	13 patients recruited; 11/13 (85%) with confirmed tumors	Feasible for tumor assessment; good concordance with high-field MRI for clinically relevant lesions	ULF-MRI identified tumor lesions in 11/11 (100%) patients compared with HF-MRI; complete lesion identification in 7/11 (63.6%); 3/4 additional relevant findings detected

## 3 Opportunities and technical trade-offs

The clinical promise of portable neuroimaging is already being explored across diverse low resource settings. Pilot deployments have occurred in parts of Africa, South Asia, and Latin America, targeting conditions such as hydrocephalus, stroke, traumatic brain injury, and pediatric infections (17–20). In Uganda, for example, low field MRI has been used to evaluate children with hydrocephalus, offering radiation free imaging where CT had previously been the only option (21). Similar initiatives have demonstrated feasibility in settings where conventional MRI has never been available. However, these benefits must be balanced against technical limitations. Ultra-low field MRI produces lower spatial resolution and signal to noise ratio than standard 1.5 or 3 Tesla scanners; scan times are longer, and advanced sequences such as high-resolution angiography or contrast enhanced studies are currently limited (14, 22, 23). Subtle lesions may be missed, and image interpretation requires adjusted expectations (14, 22, 23).

However, in many underserved contexts, the relevant comparison is not between low-field and high-field MRI, but between low-field MRI and no imaging at all. For major pathologies such as intracranial hemorrhage, large ischemic strokes, hydrocephalus, mass lesions, and gross structural abnormalities, portable MRI has shown adequate diagnostic performance (12, 13, 17, 18, 24). Ongoing advances in AI based reconstruction and denoising are further improving image quality, narrowing the practical gap with conventional systems (25–27). Rather than replacing high-field MRI, portable neuroimaging is best understood as a component of a tiered diagnostic ecosystem. Within this framework, its primary role is to enable decentralized triage, early detection, longitudinal monitoring, and risk management at lower levels of care, while preserving referral pathways for advanced imaging and specialist evaluation. This model aligns with the operational realities of LMIC health systems and represents a shift from technology-centric adoption to system-level integration aimed at maximizing the impact at the population level.

## 4 Artificial intelligence as a force multiplier

**Figure 1:**
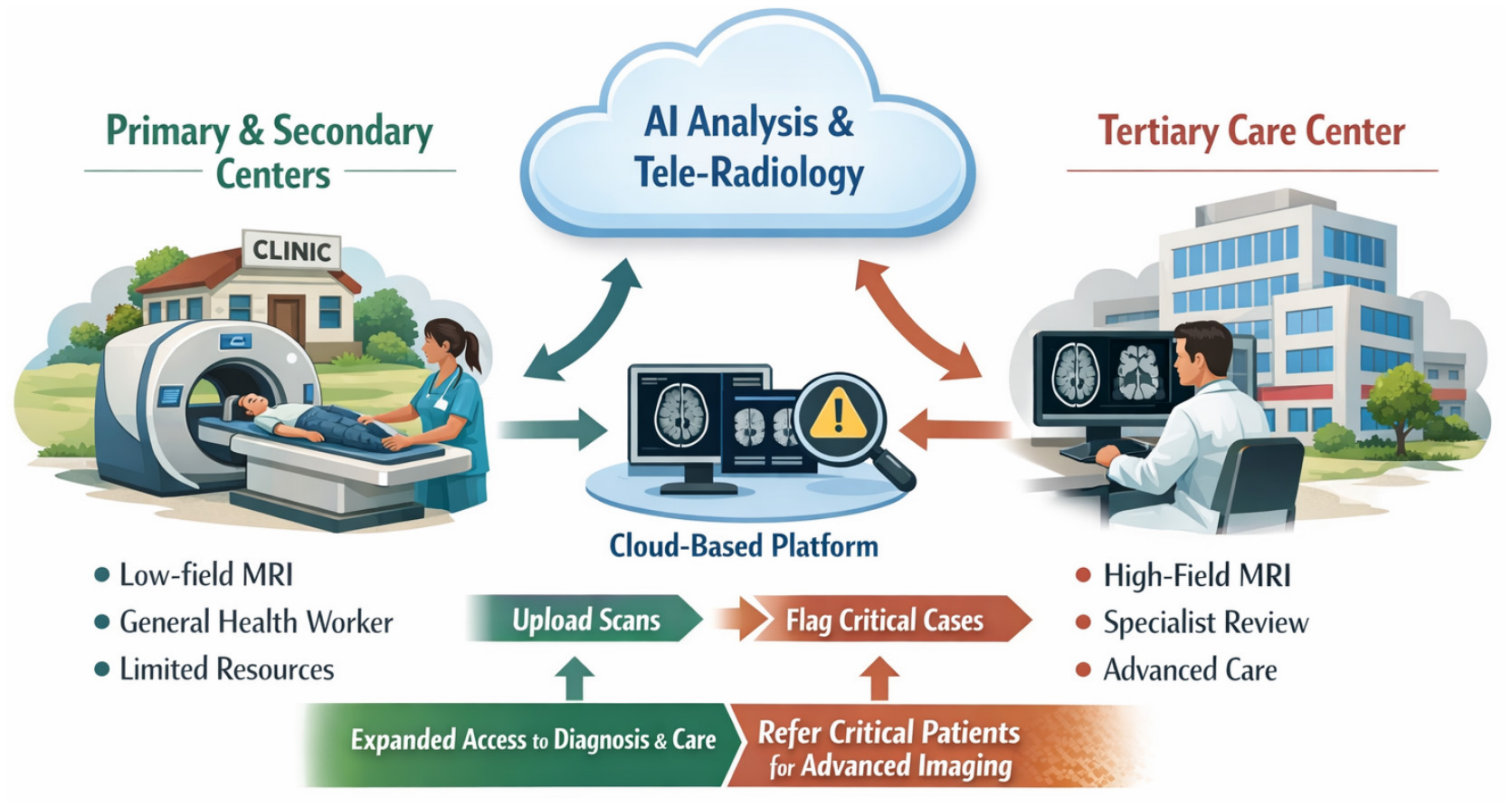
Conceptual framework illustrating a hybrid neuroimaging care pathway integrating low field portable MRI and artificial intelligence at primary and secondary care levels with specialist oversight and referral to tertiary centres.

Artificial intelligence is integral to the viability and scalability of portable neuroimaging. At the acquisition level, AI driven reconstruction algorithms enhance image quality in real time, compensating for the physical constraints of low magnetic field strength (25–27). Deep learning based denoising, artifact correction, and super resolution techniques have transformed previously marginal images into clinically interpretable scans (25–27). Beyond reconstruction, AI offers the potential to democratize image interpretation. Automated algorithms can detect and highlight abnormalities, prioritize urgent findings, and provide preliminary diagnostic suggestions (28). Multiple studies have shown that AI systems can identify strokes, hemorrhages, and space occupying lesions with accuracy comparable to expert radiologists under defined conditions (29–33). The World Health Organization has recognized AI as a potential tool to mitigate global shortages of diagnostic specialists, particularly in imaging intensive fields or in settings where specialists are scarce (34). I want to be clear, I'm not advocating for a replacement of radiologists or neuroradiologist by AI and low-field systems handled by less personnel; rather, the use of AI and low-field systems should be reserved for primary or even secondary centers, with limited resolution capacity, economic resources and accessibility constraints with clear guidelines to further direct selected patients to tertiary care where they can receive proper high-field imaging (when required) interpreted by a specialist. In this manner, we can save both resources and decrease public expenditure while also incrementing the access to proper diagnosis and care to areas where this is non-existent and considered a luxury. In practical terms, AI enables a model in which a portable scanner operated by general health workers can be paired with automated decision support and remote specialist oversight. Scans acquired in rural clinics can be uploaded to cloud-based platforms, where AI performs initial analysis and flags critical cases for tele-radiology review or referrals to tertiary centers with resolution capabilities; this hybrid model preserves clinical accountability while dramatically extending specialist reach. In Figure 1, I propose a potential conceptual framework of how I envision these hybrid application of AI assisted tele-radiology.

Nevertheless, responsible deployment is essential. Algorithms and guidelines must be validated on local populations and low-field data to avoid performance degradation. Clinicians must be trained to understand AI outputs, including their limitations, and governance frameworks must address data privacy, bias, and accountability. When implemented thoughtfully, AI does not replace clinicians but amplifies their capacity, making advanced diagnostics feasible where they were previously unattainable; however, while AI has demonstrated performance comparable to expert radiologists under controlled conditions, its deployment in real-world, low-resource settings requires careful validation, as differences in imaging quality, population characteristics, and disease patterns may affect performance and reliability (29–33). A summary of major AI applications in neuroimaging and their principal limitations is provided in Table 2.

**Table 2:** Major artificial intelligence applications in neuroimaging and key limitations.

Application	Function	Clinical relevance	Key limitations
AI-based image reconstruction	Denoising, super-resolution	Improves low-field MRI interpretability	Requires high-quality training datasets, may introduce artifacts
Automated stroke detection	Identification of ischemia/hemorrhage	Enables rapid triage and prioritization	Performance varies across populations and imaging modalities
Tumor detection and classification	Lesion segmentation and differential diagnosis	Supports clinical decision-making	Limited generalisability, risk of overfitting
Workflow prioritization algorithms	Flagging urgent scans	Reduces time to treatment	Integration challenges in low-resource systems
Tele-radiology AI triage	Pre-screening for remote interpretation	Expands specialist reach	Data privacy, infrastructure dependency, regulatory concerns

## 5 Public health impact across levels of prevention

Within this proposed decentralized neuroimaging framework, the public health relevance of portable MRI and AI extends across all levels of prevention, linking diagnostic accessibility with population-level disease control strategies. At the level of primary prevention, expanded access to imaging can reveal silent or early pathology that informs risk reduction strategies. Detection of asymptomatic lesions, early vascular changes, or regional disease patterns can guide targeted interventions and public health policies (35, 36). In endemic areas, imaging data may highlight preventable causes of neurological disease such as parasitic infections or poorly controlled hypertension, strengthening the case for upstream interventions (35, 37, 38).

Secondary prevention represents the most immediate impact. Early diagnosis is critical in conditions such as stroke, traumatic brain injury, central nervous system infections, and epilepsy; delays in imaging are a major contributor to preventable disability and death in LMICs (39–41). Portable MRI deployed in regional centers or emergency settings could shorten diagnostic timelines, enabling timely thrombolysis, surgical referral, or antimicrobial treatment. In epilepsy, access to imaging can identify surgically treatable lesions that would otherwise remain undetected, transforming lifelong disease trajectories (41). Tertiary prevention is equally important; chronic neurological conditions require ongoing monitoring to prevent complications and deterioration. Portable imaging facilitates follow-up for stroke survivors, post-surgical patients, and children with congenital conditions, reducing dependence on distant tertiary hospitals. Earlier detection of complications such as shunt malfunction, recurrent tumors, or silent infarcts allows intervention before irreversible harm occurs (42–44). By embedding portable and easily accessible imaging into longitudinal care, health systems could potentially reduce disability, improve quality of life, and lower long-term costs.

### 5.1 Life-course and equity perspective

The incorporation of portable neuroimaging and AI into healthcare systems requires an understanding within a life-course and equity framework, wherein access to diagnostic tools affects disease trajectories from early childhood to advanced age. In pediatric populations, conditions like hydrocephalus require repeated imaging for diagnosis and follow-up; however, access to MRI is often restricted in many low-resource settings, where CT frequently represents the sole available modality, if any (18, 21). Portable MRI provides a radiation-free and more accessible option, facilitating safer longitudinal monitoring and earlier intervention. In epilepsy, which affects more people in low- and middle-income countries than in high-income countries, limited access to neuroimaging leads to missed diagnoses and delays in finding surgically treatable lesions, that if operated upon, would result in a reduction of years lived with disability (41).

In adulthood, prompt diagnosis of acute neurological conditions, including stroke and traumatic brain injury, is essential to prevent disability and mortality; however, delays in imaging is still a significant obstacle in resource-limited environments (39, 40). Portable MRI used at secondary or regional centers could speed up the process of making a diagnosis and help doctors decide on treatment sooner. In later life, imaging is important for finding silent cerebrovascular disease and keeping an eye on neurodegenerative processes that lead to cognitive decline (42). Detecting these conditions sooner may allow for preventive measures and lessen the long-term strain on healthcare systems. Certainly, structural determinants of health have a big impact on these life-course trajectories. The availability of neuroimaging in LMICs is heavily affected by how the health system is funded, how infrastructure is spread out, and how many workers are available; most services are located in urban tertiary centers (6, 9). Because of this, people living in rural areas, indigenous communities, and other marginalized groups have to deal with significant issues like long travel times, high out-of-pocket costs, and long wait times for referrals (16). Portable MRI breaks down these barriers by spreading out imaging capacity, which makes it possible to get diagnostic services closer to where care is needed. When used with AI-assisted interpretation and telemedicine networks, this method can help with the lack of specialists and make neurological diagnostics more accessible across regions (34).

In my opinion, portable neuroimaging and AI are not merely technological advancements; they are tools capable of transforming neurological care pathways throughout the lifespan, especially in contexts where systemic inequities have traditionally hindered access to prompt diagnosis and treatment.

## 6 Policy and implementation considerations

Technology alone is insufficient to achieve equitable impact. Successful integration of portable neuroimaging and AI requires supportive policy frameworks, sustainable financing, and workforce development; governments and donors should explore acquisition models that lower entry barriers for public hospitals, including pooled purchasing, leasing, or pay per scan arrangements. Training programs must equip general clinicians and nurses with the skills to operate devices and interpret results within defined protocols that prioritize patients' safety. Certainly, interoperability and referral pathways are critical. Portable imaging must be linked to telemedicine networks and referral centers to ensure continuity of care (Figure 1). Maintenance, quality assurance, and data governance should be planned from the outset, although the simplified design of low-field systems reduces many traditional barriers.

Skepticism regarding image quality and implementation feasibility is therefore justified. While early experience suggests that clinically meaningful benefits can be achieved with images that are “good enough” rather than perfect (45), successful deployment depends on careful integration into health systems, including training, maintenance, regulatory alignment, and clear referral pathways. In underserved settings, the ethical considerations often favor wider access over maximal technical sophistication; however, this must be balanced against the risks of inappropriate use, over-reliance on automated systems, and unequal distribution of benefits.

Regardless of their promise, the implementation of portable neuroimaging and AI is associated with several important limitations that must be critically considered. First, algorithmic bias remains a significant concern, as many AI models are trained on datasets originated from high-income settings and high-field imaging, which may limit their generalizability to low-field data and diverse populations (25–27, 34). Without local validation, there is a risk of reduced diagnostic accuracy or systematic misclassification in underrepresented groups. Second, sustainability and maintenance pose practical challenges; although portable MRI reduces infrastructure requirements, devices still require specialized technical support, quality assurance, and reliable supply chains, which may be difficult to ensure in resource-limited settings (6, 9). Third, cost-effectiveness must be interpreted cautiously. While acquisition costs are lower than conventional MRI, long-term expenses related to maintenance, training, data storage, and connectivity may affect initial savings, particularly in fragmented health systems (5, 6). Fourth, regulatory and reimbursement frameworks in many LMICs are not yet adapted to support AI-assisted diagnostics or decentralized imaging models, potentially limiting scalability and integration into national health systems (34). Finally, there is a risk of over-reliance on AI in settings with limited specialist oversight; while AI can support decision-making, unregulated dependence without adequate training or referral pathways may lead to diagnostic errors or delayed referral of complex cases. For example, implementation experiences in resource-constrained settings such as Malawi have demonstrated that, although portable MRI can be successfully integrated into clinical workflows, challenges related to infrastructure, training, and maintenance remain important considerations for scalability (19). Therefore, proper training, implementation protocols, oversight and regulatory control are necessary to properly implement the suggested model.

## 7 Conclusion

This opinion proposes a health system-oriented framework in which portable neuroimaging and artificial intelligence are integrated into a tiered, decentralized model of neurological care; by aligning technological innovation with levels of prevention and referral pathways, this approach shifts the focus from isolated diagnostic capability to equitable system design. In doing so, it provides a conceptual basis for policymakers and clinicians to implement scalable solutions that address longstanding disparities in access to brain imaging and neurological care in LMICs. The potential impact of this model spans prevention, from early risk identification to timely diagnosis and long-term disease management. In Latin America and similar regions, where neurological disease burden is high and specialist resources are unevenly distributed, portable MRI and AI supported diagnostics can strengthen health system resilience and responsiveness; they transform advanced neuroimaging from a centralized luxury into a scalable public good. However, realizing this potential will require coordinated action from clinicians, researchers, policymakers, and industry. Technical limitations, training needs, and governance challenges must be addressed transparently; yet the direction of travel is clear. Portable neuroimaging and AI have given global health the tools to extend the frontiers of neurological care and the task now is to deploy them wisely, inclusively, and with a focus on population level benefit, ensuring that advances in brain diagnostics serve not only the few, but the many, moving away from hard-to fulfill diagnostic requirements that are often only possible in resource-rich settings with access to cutting edge technology.

## References

[1] Hricak H Abdel-Wahab M Atun R Lette MM Paez D Brink JA . Medical imaging and nuclear medicine: a lancet oncology commission. Lancet Oncol. (2021) 22:e136–72. doi: 10.1016/S1470-2045(20)30751-8 33676609 PMC8444235

[2] Parag P Hardcastle TC. Shortage of radiologists in low to middle income countries in the interpretation of CT scans in trauma. Bangladesh J Med Sci. (2022) 21:489–91. doi: 10.3329/bjms.v21i3.59560

[3] Geethanath S Vaughan JT. Accessible magnetic resonance imaging: a review. J Magn Reson Imaging JMRI. (2019) 49:e65–77. doi: 10.1002/jmri.26638 30637891

[4] World Health Organization. Global atlas of medical devices [Internet] (2017). Available online at: https://iris.who.int/items/86c5fc07-a7cb-44fb-a21d-373b18613b6e (Accessed January 12, 2026).

[5] Altaf A Shakir M Irshad HA Atif S Kumari U Islam O . Applications, limitations and advancements of ultra-low-field magnetic resonance imaging: a scoping review. Surg Neurol Int. (2024) 15:218. doi: 10.25259/SNI_162_2024 38974534 PMC11225429

[6] Murali S Ding H Adedeji F Qin C Obungoloch J Asllani I . Bringing MRI to low- and middle-income countries: directions, challenges and potential solutions. NMR Biomed. (2024) 37:e4992. doi: 10.1002/nbm.4992 37401341

[7] Fornari A Lanza M Guastafierro E Marcassoli A Sismondo P Curatoli C . Inequities in neurological care: access to services, care gaps, and other barriers—a systematic review. Eur J Neurol. (2024) 32:e16553. doi: 10.1111/ene.16553 39582360 PMC11625953

[8] Clement David-Olawade A Olawade DB Vanderbloemen L Rotifa OB Fidelis SC Egbon E . AI-driven advances in low-dose imaging and enhancement—a review. Diagnostics. (2025) 15:689. doi: 10.3390/diagnostics15060689 40150031 PMC11941271

[9] Scott AM Ngwa W Hricak H. Strengthening medical imaging capacity will save lives. Nat Med. (2025) 31:3954–5. doi: 10.1038/s41591-025-04007-9 41087564

[10] Sarkodie BD Ohene-Botwe B Mensah YB Tagoe E Jimah BB Brakohiapa EK . Density and regional distribution of radiologists in a low-income country: the Ghana situation. Chin J Acad Radiol. (2023) 6:188–95. doi: 10.1007/s42058-023-00130-z

[11] Frija G BlaŽić I Frush DP Hierath M Kawooya M Donoso-Bach L . How to improve access to medical imaging in low- and middle-income countries ? eClinMed [Internet]. (2021) 38. Available online at: https://www.thelancet.com/journals/eclinm/article/PIIS2589-5370(21)00314-X/fulltext?utm_source=chatgpt.com (Accessed January 13, 2026). doi: 10.1016/j.eclinm.2021.101034 PMC8318869 34337368

[12] von Danwitz NM Lehnen NC Meißner JN Samani OS Asperger H Thielscher C . Portable ultra-low-field MRI in acute stroke care: a pilot study. Eur Stroke J. (2025) 10:1430–7. doi: 10.1177/23969873251344761 40515383 PMC12170540

[13] Mazurek MH Cahn BA Yuen MM Prabhat AM Chavva IR Shah JT . Portable, bedside, low-field magnetic resonance imaging for evaluation of intracerebral hemorrhage. Nat Commun. (2021) 12:5119. doi: 10.1038/s41467-021-25441-6 34433813 PMC8387402

[14] Kravchenko D Hagar MT Vecsey-Nagy M Kabat I Groteklaes A Luetkens JA . Low-field and portable MRI technology: advancements and innovations. Eur Radiol Exp. (2025) 9:103. doi: 10.1186/s41747-025-00638-2 41129051 PMC12549457

[15] Swoop^®^ Portable MRI System | AI-Powered Brain Imaging [Internet]. Available online at: https://hyperfinemri.com/homepage-ous (Accessed January 13, 2026).

[16] Ruano AL Rodríguez D Rossi PG Maceira D. Understanding inequities in health and health systems in Latin America and the Caribbean: a thematic series. Int J Equity Health. (2021) 20:94. doi: 10.1186/s12939-021-01426-1 33823879 PMC8023548

[17] Shoghli A Chow D Kuoy E Yaghmai V. Current role of portable MRI in diagnosis of acute neurological conditions. Front Neurol. (2023) 14:1255858. doi: 10.3389/fneur.2023.1255858 37840918 PMC10576557

[18] Gruber MD Unadkat P Morales DM Joshi S Limbrick DD Mittler MA . Ultra-low-field portable MRI for assessing ventricular size in pediatric hydrocephalus: a feasibility study. J Neurosurg Pediatr. (2025) 36:11–9. doi: 10.3171/2025.1.PEDS24358 40250047

[19] Chetcuti K Chilingulo C Goyal MS Vidal L O'Brien NF Postels DG . Implementation of a low-field portable MRI scanner in a resource-constrained environment: our experience in Malawi. AJNR Am J Neuroradiol. (2022) 43:670–4. doi: 10.3174/ajnr.A7494 35450856 PMC9089250

[20] Obungoloch J Muhumuza I Teeuwisse W Harper J Etoku I Asiimwe R . On-site construction of a point-of-care low-field MRI system in Africa. NMR Biomed. (2023) 36:e4917. doi: 10.1002/nbm.4917 36914258 PMC10330026

[21] TU Delft [Internet]. A sustainable MRI system to diagnose hydrocephalus in Uganda. Available online at: https://www.tudelft.nl/en/diagnostics-for-all/projects/a-sustainable-mri-system-to-diagnose-hydrocephalus-in-uganda (Accessed January 13, 2026).

[22] Gagliardo C Feraco P Contrino E D'Angelo C Geraci L Salvaggio G . Ultra-low-field MRI: a David versus Goliath challenge in modern imaging. Radiol Med (Torino). (2025) 130:2012–29. doi: 10.1007/s11547-025-02091-y 41003941 PMC12669372

[23] Ladd ME Bachert P Meyerspeer M Moser E Nagel AM Norris DG . Pros and cons of ultra-high-field MRI/MRS for human application. Prog Nucl Magn Reson Spectrosc. (2018) 109:1–50. doi: 10.1016/j.pnmrs.2018.06.001 30527132

[24] Zeyen T Sabir H Bauer T Henke O Lehnen N Zidan M . Proof of concept: portable ultra-low-field MRI for the assessment of brain tumors. Neuro-Oncol Pract. (2025) npaf101. doi: 10.1093/nop/npaf101 PMC13153708 42111036

[25] Islam KT Zhong S Zakavi P Chen Z Kavnoudias H Farquharson S . Improving portable low-field MRI image quality through image-to-image translation using paired low- and high-field images. Sci Rep. (2023) 13:21183. doi: 10.1038/s41598-023-48438-1 38040835 PMC10692211

[26] Ssentamu T Kimbowa A Omoding R Atamba E Mukwaya PK Jjuuko GW . Denoising very low-field magnetic resonance images using native noise modeling. Front Neuroimaging [Internet]. (2025). Available online at: https://www.frontiersin.org/journals/neuroimaging/articles/10.3389/fnimg.2025.1501801/full (Accessed January 13, 2026). doi: 10.3389/fnimg.2025.1501801 PMC12089061 40395481

[27] Islam KT Zhong S Zakavi P Kavnoudias H Farquharson S Durbridge G . AI improves consistency in regional brain volumes measured in ultra-low-field MRI and 3T MRI. Front Neuroimaging [Internet]. (2025). Available online at: https://www.frontiersin.org/journals/neuroimaging/articles/10.3389/fnimg.2025.1588487/full (Accessed January 13, 2026). doi: 10.3389/fnimg.2025.1588487 PMC12174951 40534653

[28] Topol EJ. High-performance medicine: the convergence of human and artificial intelligence. Nat Med. (2019) 25:44–56. doi: 10.1038/s41591-018-0300-7 30617339

[29] Cè M Irmici G Foschini C Danesini GM Falsitta LV Serio ML . Artificial intelligence in brain tumor imaging: a step toward personalized Medicine. Curr Oncol. (2023) 30:2673–701. doi: 10.3390/curroncol30030203 36975416 PMC10047107

[30] Titano JJ Badgeley M Schefflein J Pain M Su A Cai M . Automated deep-neural-network surveillance of cranial images for acute neurologic events. Nat Med. (2018) 24:1337–41. doi: 10.1038/s41591-018-0147-y 30104767

[31] Soun JE Chow DS Nagamine M Takhtawala RS Filippi CG Yu W . Artificial intelligence and acute stroke imaging. AJNR Am J Neuroradiol. (2021) 42:2–11. doi: 10.3174/ajnr.A6883 33243898 PMC7814792

[32] Matsumoto R Matsuo H Sugimoto M Matsunaga T Nishio M Kono AK . Deep learning-based detection of intracranial hemorrhages in postmortem computed tomography: comparative study of 15 transfer-learned models. Appl Sci. (2025) 15:10513. doi: 10.3390/app151910513

[33] Rauschecker AM Rudie JD Xie L Wang J Duong MT Botzolakis EJ . Artificial intelligence system approaching neuroradiologist-level differential diagnosis accuracy at brain MRI. Radiology. (2020) 295:626–37. doi: 10.1148/radiol.2020190283 32255417 PMC7263320

[34] Ethics and governance of artificial intelligence for health [Internet]. Available online at: https://www.who.int/publications/i/item/9789240029200 (Accessed January 13, 2026).

[35] Moyano LM O'Neal SE Ayvar V Gonzalvez G Gamboa R Vilchez P . High prevalence of asymptomatic neurocysticercosis in an endemic rural community in Peru. PLoS Negl Trop Dis. (2016) 10:e0005130. doi: 10.1371/journal.pntd.0005130 27992429 PMC5167259

[36] Santulli G Savino M Komici K Mone P Savino L Jankauskas SS. A novel imaging marker for asymptomatic cerebrovascular lesions in hypertension. Am J Hypertens. (2024) 37:859–60. doi: 10.1093/ajh/hpae100 39094226 PMC11471834

[37] Del Brutto OH Peinado CD Mera RM Del Brutto VJ Sedler MJ. Neuroimaging signatures of cerebral small vessel disease and risk of falls in stroke-free older adults living in rural Ecuador. Atahualpa Project J Neurol Sci. (2019) 402:133–5. doi: 10.1016/j.jns.2019.05.019 31132535

[38] Del Brutto OH Mera RM Viteri EM Pólit J Ledesma EA Cano JA . Hypertensive retinopathy and cerebral small vessel disease in Amerindians living in rural Ecuador: the Atahualpa project. Int J Cardiol. (2016) 218:65–8. doi: 10.1016/j.ijcard.2016.05.020 27232913

[39] Buque H Smith L Lopes D Pizzol D Lorenzo E Arroz N . Delays in the stroke care pathway in a low-income setting: an audit study from Mozambique. Int J Environ Res Public Health. (2025) 22:1008. doi: 10.3390/ijerph22071008 40724075 PMC12294557

[40] Wiyarta E Fisher M Kurniawan M Hidayat R Geraldi IP Khan QA . Global insights on prehospital stroke care: a comprehensive review of challenges and solutions in low- and middle-income countries. J Clin Med. (2024) 13:4780. doi: 10.3390/jcm13164780 39200922 PMC11355367

[41] Sen A Newton CR Ngwende G. Epilepsy in low- to middle-income countries. Curr Opin Neurol. (2025) 38:121–7. doi: 10.1097/WCO.0000000000001350 39927414 PMC11888831

[42] Vermeer SE Prins ND den Heijer T Hofman A Koudstaal PJ Breteler MMB. Silent brain infarcts and the risk of dementia and cognitive decline. N Engl J Med. (2003) 348:1215–22. doi: 10.1056/NEJMoa022066 12660385

[43] Abdalla G Hammam A Anjari M D'Arco DF Bisdas DS. Glioma surveillance imaging: current strategies, shortcomings, challenges and outlook. BJR|Open. (2020) 2:20200009. doi: 10.1259/bjro.20200009 33178973 PMC7594888

[44] Hersh DS Kumar R Klimo P Bookland M Martin JE. Hydrocephalus surveillance following shunt placement or endoscopic third ventriculostomy: a survey of surgeons in the hydrocephalus clinical research networks. J Neurosurg Pediatr. (2021) 28:139–46. doi: 10.3171/2020.12.PEDS20830 34020413

[45] Kimberly WT Sorby-Adams AJ Webb AG Wu EX Beekman R Bowry R . Brain imaging with portable low-field MRI. Nat Rev Bioeng. (2023) 1:617–30. doi: 10.1038/s44222-023-00086-w 37705717 PMC10497072

